# Supplementary material for: Proliferation Drives Aging-Related Functional Decline in a Subpopulation of the Hematopoietic Stem Cell Compartment
Source: Cell Rep. 2017 May 23;19(8):1503–11. doi: 10.1016/j.celrep.2017.04.074 (PMC5457484; doi:10.1016/j.celrep.2017.04.074)
Supplement: Document S1. Supplemental Experimental Procedures and Figures S1 and S2 [file mmc1.pdf]

Cell Reports, Volume *19*

Supplemental Information

Proliferation Drives Aging-Related Functional Decline in a Subpopulation of the Hematopoietic Stem Cell Compartment

Kristina Kirschner, Tamir Chandra, Vladimir Kiselev, David Flores-Santa Cruz, Iain C. Macaulay, Hyun Jun Park, Juan Li, David G. Kent, Rupa Kumar, Dean C. Pask, Tina L. Hamilton, Martin Hemberg, Wolf Reik, and Anthony R. Green

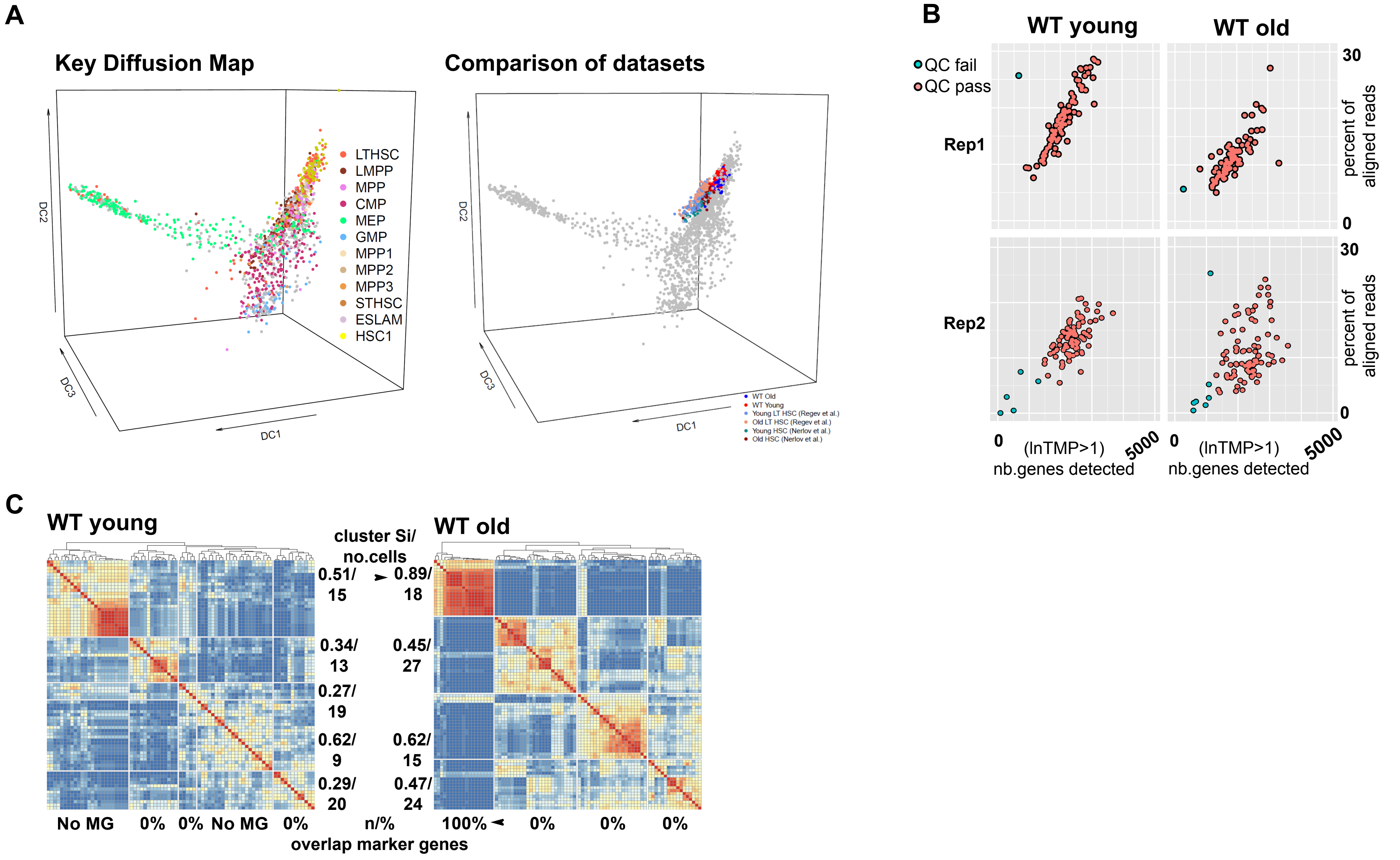

Supplemental Figure 1: Additional quality control and analysis of single cell RNA-Seq data, Related to Figure 1:
A) Diffusion map defining the single cell transcriptome of several murine haematopoietic cell types as indicated on left hand side (Nestorowa et al., 2016). Our HSC data alongside the HSC data from Grover and colleagues (Grover et al., 2016) and Regev group (Kowalczyk et al., 2015) were then superimposed onto the diffusion map (grey cells, right hand side). Colours indicate the cell types on left hand side as specified in the legend and data sets on right hand side. B) Quality control plots showing individual cells failing (green) and passing (red) the test. The percentage of aligned reads over the number of genes detected is shown with a cut off of 1300 genes. TMP transcript per million. C) Heat maps of all young or all old HSCs using SC3 unsupervised clustering tool. The intensity of the cluster is indicated from 0 (blue, not upregulated) to 1 (red, upregulated). The silhouette index (si) is given for each cluster. The overlap with marker genes identified in Fig.1c is given as percentage of all cells analysed for each age group. No MG denotes a lack of marker genes for a given cluster.

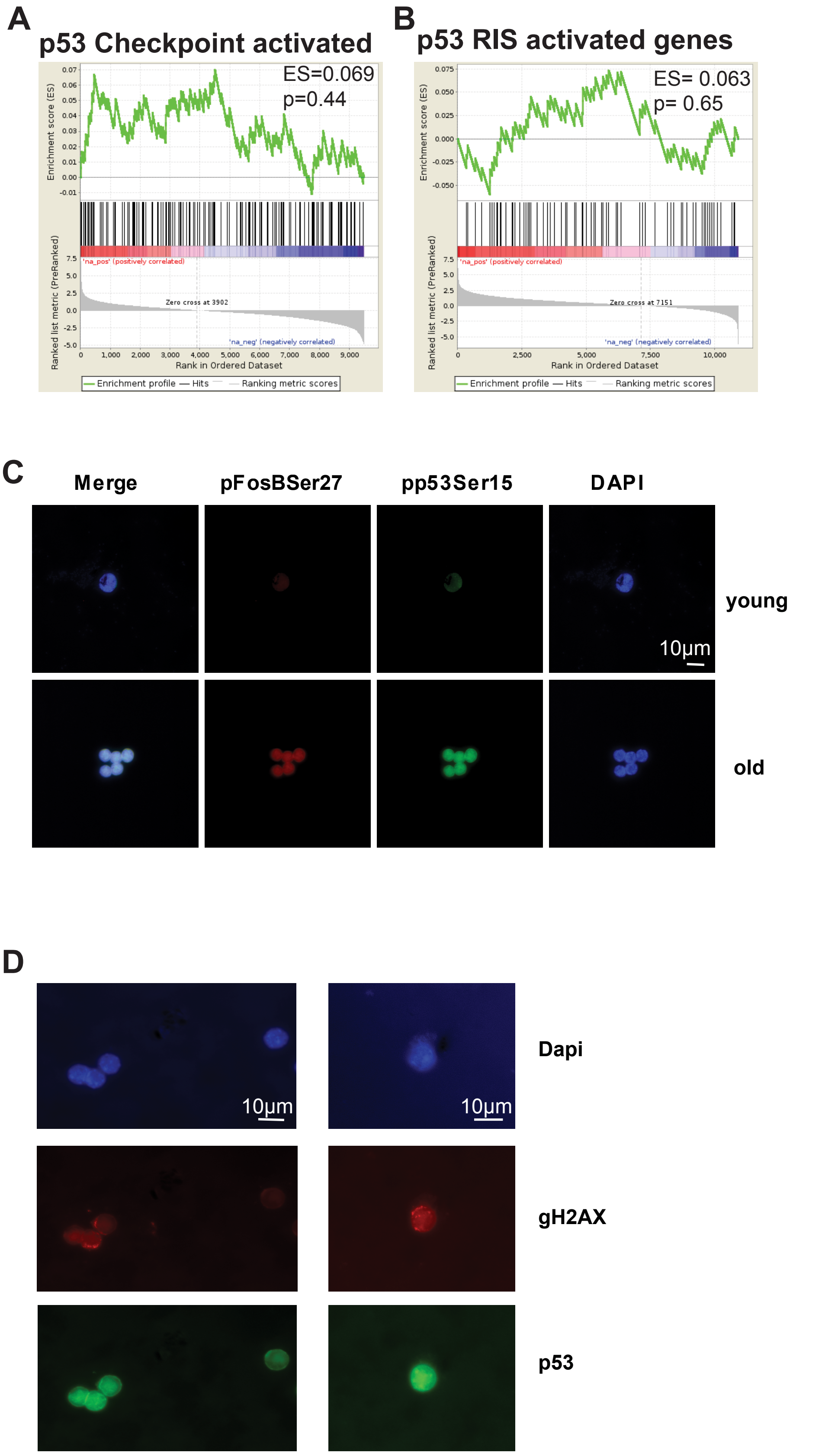

Supplemental Figure 2: Additional data supporting p53 upregulation in old specific cluster only, Related to Figure 2:
A) Gene Set enrichment plot (Subramanian et al., 2005) comparing p53 checkpoint related target genes with differentially expressed genes between the old specific cluster and all other old HSCs. Adjusted p-value and enrichment score are indicated. B) Gene Set enrichment plot (Subramanian et al., 2005) comparing p53 RIS target genes with differentially expressed genes between pooled young and old WT HSCs. Adjusted p-value and enrichment score are indicated. C) Immunofluorescence images of young and old HSCs stained for p53 phosphorylated at Serine 15 (green) and gH2AX phosphorylated at Serine 139 (red). DAPI stained nuclei are blue. Representative images are shown. D) Immunofluorescence images of old HSCs stained for p53 phosphorylated at Serine 15 (green) and FosB phosphorylated at Serine 27 (red). DAPI stained nuclei are blue. Representative images are shown.

Supplemental methods

Flow cytometry

All samples were index sorted on a BD Influx with filters and five lasers. Single ESLAM cells were either sorted into round bottom 96 well plates preloaded with serum-free medium for kinetics experiments or into 96 well PCR plates containing a mild lysis buffer with ribonuclease inhibitor as described for single cell RNA-Sequencing (Picelli et al., 2013). For Immunofluorescence experiments, ESLAM cells were sorted into tubes and mounted onto coated Cytoslides (Thermo Scientific).

Data analysis

96 single cells samples were sequenced, returning a variable number of reads (μ = 2, 017, 758 σ = 1, 886, 443). RNA-Seq by Expectation Maximisation) (RSEM) (Li and Dewey, 2011) which implements an Expectation Maximisation algorithm that allows computing the maximum likelihood of the estimate by combining information from both unique and multi mapped reads was used to estimate number of transcripts and transcripts per million (TPM) per gene. Parallelization of the alignment was accomplished with GNU Parallel ("GNU Parallel: The Command-Line Power Tool | USENIX," n.d.). The reads were mapped against the Ensembl mouse reference genome version GRC3877 (Kersey et al., 2016) with the inclusion of the reference for the spike in controls from the ERCC consortium (Baker et al., 2005) using Tophat (Trapnell et al., 2009). TPM estimates were used for quality control of the cells similarly to Kumar and colleagues (Kumar et al., 2014). Cells with less than 2500 detected transcripts were removed from posterior analysis. The SCDE R package from Kharchenko Lab (Kharchenko et al., 2014) provides a method for the analysis of differential expression in single cells. Estimate counts per gene previously calculated with RSEM (Li and Dewey, 2011) were used to compute the differential expression, genes with insufficient number of reads or not seen in enough cells were removed. Individual posterior shows the likelihood of differential expression for each cell. In Figure 1F) SCDE plots for marker genes of cluster 1 in young (blue) and old (orange) HSCs are shown. Expression levels of individual cells are given by individual lines. Individual posterior shows the likelihood of differential expression for each cell. Joint posterior depicts the likelihood of differential expression of a group of cells within the same condition. The black line indicates the estimated joint posterior distribution for the overall level for each cell type. MLE denotes the likelihood of differential expression between two conditions and adjusted Z-scores are given for indicated genes comparing two conditions.

RNA-Seq data were clustered and marker genes obtained by using SC3 (Kiselev et al., 2016). KEGG pathway analysis (Kanehisa et al., 2012) was performed using Webgestalt (Wang et al., 2013). Entrez Genes were annotated and pathway analysis conducted using hypergeometric test with multiple test adjustment (BH) and significance level of Top10. A minimum of 2 genes per category needed to be present. Enrichment analysis was performed using Gene Set Enrichment Analysis (GSEA) (Subramanian et al., 2005). The z-score of the expression difference provided by the SCDE package (Kharchenko et al., 2014) between old specific and all other old WT HSCs (p53, Stat3, Stat5, TPO and Lineage markers, see Figure 2C and 2G) or between young WT and Jak2V617F HSCs was used as a rank list of genes to compare against a defined set of genes as a background (p53, Stat3, Stat5, Sup. Fig. 2B). The significance of the overlap between marker genes and p53 targets or Stat3 and Stat5 was calculated using the hypergeometric test. The p-value was calculated assuming a total of m white balls and 20000-m black balls where a total of 20 balls were drawn. Here, m is either 313 or 768 and the number of white balls obtained was either 6 or 2.

For microarray analysis, raw probe-set intensities were processed with the R package lumi (Du et al., 2008). The lumiR function allows the processing of the data with the package, while mapping the Illumina identifiers to Mus musculus RefSeq IDs and Entrez_gene_ID through the lumi MouseID Mapping package. The raw data was normalised with the lumiExpresso function (Du et al., 2008). The differentially expressed genes were identified using the limma package (Smyth, 2004).

Supplemental References

Alder, J.K., Georgantas, R.W., Hildreth, R.L., Civin, C.I., 2006. 348. Kruppel-Like Factor 4 Regulates Proliferation of Human and Mouse Hematopoietic Stem-Progenitor Cells, but Is Not Essential for Mouse Hematopoietic Repopulation. Mol. Ther. 13, S132–S133. doi:10.1016/j.ymthe.2006.08.406

Berger, I., Shaul, Y., 1998. c-Fos antagonizes the junD gene positive autoregulatory loop; a novel c-Fos role in promoter switching. Gene 211, 375–382. doi:10.1016/S0378-1119(98)00120-6

Kowalczyk, M.S., Tirosh, I., Heckl, D., Rao, T.N., Dixit, A., Haas, B.J., Schneider, R.K., Wagers, A.J., Ebert, B.L., Regev, A., 2015. Single-cell RNA-seq reveals changes in cell cycle and differentiation programs upon aging of hematopoietic stem cells. Genome Res. . doi:10.1101/gr.192237.115

Liebermann, D.A., Gregory, B., Hoffman, B., 1998. International journal of oncology., International Journal of Oncology. University of Crete, Faculty of Medicine, Laboratory of Clinical Virology.

Mayani, H., 2016. The regulation of hematopoietic stem cell populations. F1000Research 5, F1000 Faculty Rev-1524. doi:10.12688/f1000research.8532.1

Okada, S., Fukuda, T., Inada, K., Tokuhisa, T., 1999. Prolonged Expression of c-fos Suppresses Cell Cycle Entry of Dormant Hematopoietic Stem Cells. Blood 93, 816 LP-825.

Santaguida, M., Schepers, K., King, B., Sabnis, A.J., Forsberg, E.C., Attema, J.L., Braun, B.S., Passegué, E., Costa, D.B., Wagner, K., al., et, 2009. JunB protects against myeloid malignancies by limiting hematopoietic stem cell proliferation and differentiation without affecting self-renewal. Cancer Cell 15, 341–52. doi:10.1016/j.ccr.2009.02.016

Sirin, O., Lukov, G., Mao, R., Conneely, O., Goodell, M.A., 2010. The Orphan Nuclear Receptor Nurr1 Restricts the Proliferation of Hematopoietic Stem Cells. Nat. Cell Biol. 12, 1213–1219. doi:10.1038/ncb2125

Vanegas, N.-D.P., Vernot, J.-P., 2017. Loss of quiescence and self-renewal capacity of hematopoietic stem cell in an in vitro leukemic niche. Exp. Hematol. Oncol. 6, 2. doi:10.1186/s40164-016-0062-1

Yu, X., Alder, J.K., Chun, J.H., Friedman, A.D., Heimfeld, S., Cheng, L., Civin, C.I., 2006. HES1 Inhibits Cycling of Hematopoietic Progenitor Cells via DNA Binding. Stem Cells 24, 876–888. doi:10.1634/stemcells.2005-0598
