# Supplementary material for: Proliferation Drives Aging-Related Functional Decline in a Subpopulation of the Hematopoietic Stem Cell Compartment
Source: Cell Rep. 2017 May 23;19(8):1503–11. doi: 10.1016/j.celrep.2017.04.074 (PMC5457484; doi:10.1016/j.celrep.2017.04.074)
Supplement: Document S2. Article plus Supplemental Information [file mmc5.pdf]

Cell Reports

Report

Proliferation Drives Aging-Related Functional Decline in a Subpopulation of the Hematopoietic Stem Cell Compartment

Graphical Abstract

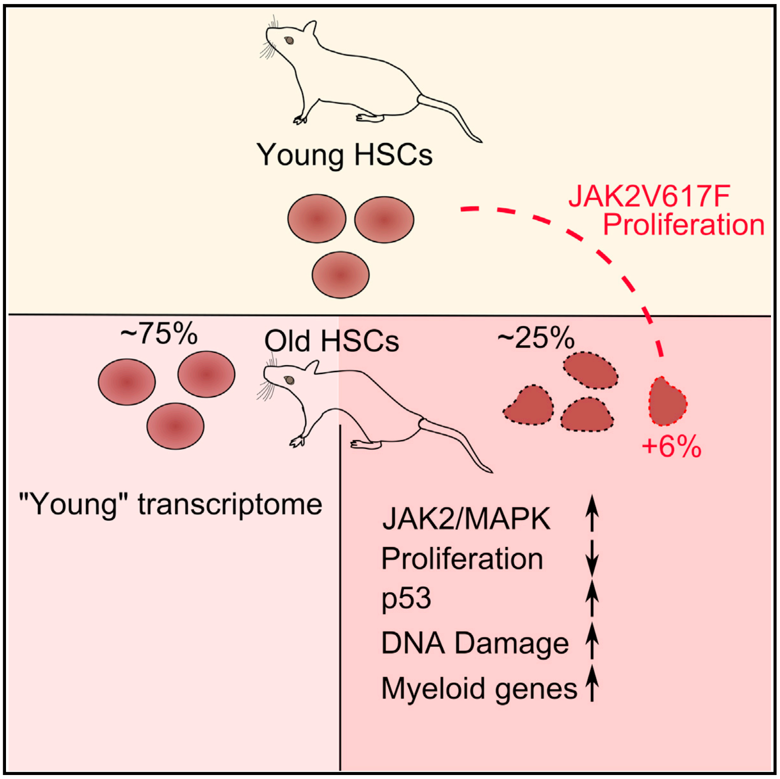

Authors

Kristina Kirschner, Tamir Chandra, Vladimir Kiselev, ..., Martin Hemberg, Wolf Reik, Anthony R. Green

Correspondence

kristina.kirschner@glasgow.ac.uk (K.K.),
tamir.chandra@igmm.ed.ac.uk (T.C.),
wolf.reik@babraham.ac.uk (W.R.),
arg100@cam.ac.uk (A.R.G.)

In Brief

Kirschner et al. describe heterogeneous aging of hematopoietic stem cells (HSCs), with a subset of old HSCs displaying signs of functional exhaustion. An increase in proliferation expands the aged HSC subgroup, linking prolonged proliferation to functional decline in HSCs.

Highlights

- Single-cell transcriptomics reveals functional decline in old HSCs
- p53-associated functional decline is driven by prolonged proliferation
- Subpopulation of HSCs show aging signature, revealing heterogeneity in the rate of aging

Accession Numbers

GSE87687
GSE87631

Kirschner et al., 2017, Cell Reports *19*, 1503–1511
May 23, 2017 © 2017 The Author(s).
http://dx.doi.org/10.1016/j.celrep.2017.04.074

Cell Reports
Report

OPEN ACCESS
CellPress

Proliferation Drives Aging-Related Functional Decline in a Subpopulation of the Hematopoietic Stem Cell Compartment

Kristina Kirschner,[1,2,3,4,*] Tamir Chandra,[5,6,*] Vladimir Kiselev,[7] David Flores-Santa Cruz,[1] Iain C. Macaulay,[7] Hyun Jun Park,[1] Juan Li,[1] David G. Kent,[1] Rupa Kumar,[1] Dean C. Pask,[1] Tina L. Hamilton,[1] Martin Hemberg,[7] Wolf Reik,[5,7,8,*] and Anthony R. Green[1,2,3,*]
[1]Cambridge Institute for Medical Research, University of Cambridge, Cambridge, Cambridgeshire CB2 0XY, UK
[2]Department of Haematology, University of Cambridge, Cambridge, Cambridgeshire CB2 0XY, UK
[3]Stem Cell Institute, University of Cambridge, Cambridge, Cambridgeshire CB2 0XY, UK
[4]Institute for Cancer Sciences, University of Glasgow, Glasgow, Lanarkshire G61 1BD, UK
[5]Epigenetics ISP, The Babraham Institute, Cambridge, Cambridgeshire CB22 3AT, UK
[6]MRC Unit for Human Genetics, University of Edinburgh, Midlothian EH2 2XU, UK
[7]The Wellcome Trust Sanger Institute, Cambridge, Cambridgeshire CB10 1SA, UK
[8]Lead Contact
*Correspondence: kristina.kirschner@glasgow.ac.uk (K.K.), tamir.chandra@igmm.ed.ac.uk (T.C.), wolf.reik@babraham.ac.uk (W.R.), arg100@cam.ac.uk (A.R.G.)
http://dx.doi.org/10.1016/j.celrep.2017.04.074

SUMMARY

Aging of the hematopoietic stem cell (HSC) compartment is characterized by lineage bias and reduced stem cell function, the molecular basis of which is largely unknown. Using single-cell transcriptomics, we identified a distinct subpopulation of old HSCs carrying a p53 signature indicative of stem cell decline alongside pro-proliferative JAK/STAT signaling. To investigate the relationship between JAK/STAT and p53 signaling, we challenged HSCs with a constitutively active form of JAK2 (V617F) and observed an expansion of the p53-positive subpopulation in old mice. Our results reveal cellular heterogeneity in the onset of HSC aging and implicate a role for JAK2V617F-driven proliferation in the p53-mediated functional decline of old HSCs.

INTRODUCTION

Organismal aging is accompanied by a gradual decline in regenerative capacities. This decline has been associated with reduced stem cell function, where the aging stem cell pool is unable to repopulate tissues upon cellular loss during physiological turnover or after tissue injury (Beerman et al., 2010). In the hematopoietic system, stem cell aging is evident in a weakening of the adaptive immune response and a general decline of hematopoietic stem cell fitness (Beerman et al., 2010).

The weakening immune response has been attributed to a shift from a balanced lymphoid/myeloid output toward a myeloid skew with age (Rossi et al., 2005). Although hematopoietic stem cells (HSCs) showing a skew in their myeloid/lymphoid output can also be found in young mice, the aggregate output is balanced. In contrast, with age, proportionally fewer lymphoid biased HSCs are found (Grover et al., 2016).

In addition to the lineage skew, aging of the hematopoietic system also results in reduced performance in blood reconstitution and engraftment, regardless of lineage output (Dykstra et al., 2011). In addition, accumulation of DNA damage and upregulation of p53 in aged HSC populations is well documented (Dumble et al., 2007; Rossi et al., 2007). p53 is a key regulator of aging in hematopoiesis, with high levels of p53 leading to premature aging features, such as reduced engraftment (Dumble et al., 2007). However, while Grover and colleagues (Grover et al., 2016) were able to shed light on the molecular signature responsible for lineage skewing with age, little is known about the molecular basis of the functional decline of HSCs with age. It is, for example, unknown how uniformly the functional impairment is distributed within the HSC compartment, and it is unclear what factors and pathways are directly relevant to the decline.

Using an index-sorting strategy and single-cell assays for highly purified long-term HSCs (LT-HSCs), we identified HSC aging as a heterogeneous process by characterizing an HSC subpopulation marked through p53 activation in old mice. Further transcriptional description of the subcluster shows myeloid bias as well as JAK/STAT- and MAPK (mitogen-activated protein kinase)-driven pro-proliferative gene signatures, reminiscent of the proliferation-driven cell-cycle arrest in cellular senescence (Serrano et al., 1997). Moreover, expansion of this old-specific subpopulation could be triggered by constitutively activating Jak2. We propose a model whereby prolonged proliferation in HSCs driven by the JAK/STAT pathway leads to a functionally impaired HSC subpopulation defined by p53 pathway upregulation with age.

CrossMark

This is an open access article under the CC BY license (http://creativecommons.org/licenses/by/4.0/).

RESULTS

The Long-Term HSC Compartment Harbors a Distinct Subpopulation with Age

To determine how the transcriptional heterogeneity in long-term HSCs is associated with age, we index-sorted single LT-HSCs using ESLAM markers (Figure 1A) from the bone marrow of mice aged 4 months old (n = 192) and 18 months old (n = 192). This approach resulted in a distinct HSC population evident through comparison with two published hematopoietic single-cell transcriptome datasets of young and old HSCs (lineage-negative Sca-1^{+}, c-Kit^{+}, CD150^{+}, and CD48^{-}) (Grover et al., 2016; Kowalczyk et al., 2015), when projecting all datasets onto an HSC expression atlas (Nestorowa et al., 2016) (Figure S1A). We obtained 119/192 old and 99/192 young cells after quality control (Figure S1B; Supplemental Experimental Procedures) and used a k-means-based consensus clustering approach for single-cell transcriptomes (SC3) (Kiselev et al., 2017).

One cluster was entirely made up of old HSCs from replicate mice (referred to as an ''old-specific'' cluster) (Figure 1B) being well defined as measured by silhouette index ([Si] 0.92; Figure 1D) and distinct. Marker genes driving cluster formation were calculated using SC3 (n = 62; Figure 1C; Table S1). To investigate whether a similar cluster exists in young LT-HSCs, cells were clustered separately (Figure S1C), with no similar cluster detectable (Figure S1C). Re-clustering old LT-HSCs separately identified the old-specific cluster with an identical subset of marker genes (Figure S1C). To ensure that differences in cell type were not driving the clustering, we compared ESLAM markers from the index sort data with no difference in intensity of CD45 ($p = 0.8925$), CD48 ($p = 0.4851$), CD150 ($p = 0.7208$), or EPCR ($p = 0.6472$) expression (Figure 1E). To validate the marker genes, we used single-cell differential expression (SCDE) (Kharchenko et al., 2014) to identify differentially expressed genes between the old-specific subpopulation and the other HSCs. SCDE confirmed a subpopulation of old LT-HSCs expressing marker genes identified by SC3 (Figure 1F). In summary, we identified a transcriptionally distinct subpopulation of old LT-HSCs.

The Old-Specific Cluster Is Characterized by Anti- and Pro-proliferative Pathways

To characterize the old-specific cluster, we characterized the marker genes underlying the clustering (Figure 1C; Table S1). One of the top marker genes, the cell-cycle regulator Cdkn1a, is a well-known p53 target (Beckerman and Prives, 2010), with p53 previously having been implicated in regulating HSC aging and quiescence (Dumble et al., 2007). To further test p53 regulation of the old-specific cluster, we used a list of senescence-specific p53 genes (Kirschner et al., 2015). We identified a significant enrichment of marker genes as p53 related (6/68 marker genes, $p = 1e{-}06$; Figure 2A; Table S1), many of which are negative regulators of proliferation. We were unable to identify enrichment of p53 apoptosis ($p = 0.5$; Kirschner et al., 2015), autophagy ($p = 0.06$; Kenzelmann Broz et al., 2013), or checkpoint ($p = 0.12$; Kenzelmann Broz et al., 2013) targets in the marker genes (Figure 2A; Table S1). No p53 targets were detected in the marker genes of the other five clusters in Figure 1C (see Table S1).

In the marker gene list, we noted multiple jun/fos transcription factors that are targets of JAK/STAT signaling in the hematopoietic system, where JAK/STAT stimulates proliferation and differentiation (Rawlings et al., 2004). To test for enrichment of JAK/STAT targets, we compared the marker genes with chromatin immunoprecipitation sequencing (ChIP-seq) data from patient megakaryocytes, where the JAK/STAT pathway drives disease (Lau et al., 2015). We identified a significant enrichment of STAT targets (Lau et al., 2015) in our marker genes (green fields in Figure 2A: $p = 0.0005$ for pStat3 targets 6/68, and $p = 0.0006$ for pStat5 targets 4/68; Table S1). No Stat3 or Stat5 targets were detected in the other five clusters in Figure 1C (Table S1). In summary, analysis of the marker genes suggested a co-activation of cell-cycle repressing p53 and pro-proliferative JAK/STAT signaling in the old-specific cluster.

Several lines of evidence are consistent with the concept that pro-proliferative and anti- proliferative stimuli exist in the same old HSC subpopulation. First, unbiased KEGG (Kyoto Encyclopedia of Genes and Genomes) pathway analysis of the marker genes (Kanehisa et al., 2012) revealed an enrichment for ''cell cycle'' due to the presence of cell-cycle inhibitors, p53 targets (Figure 2B, red bars), and the MAPK pathway (Figure 2B, green bar), which is driven by JAK activation in hematological cells (Vainchenker and Constantinescu, 2013).

Second, we compared gene target lists for p53, STAT3, and STAT5 to marker genes for the old-specific HSC cluster. Gene set enrichment analysis (GSEA) (Subramanian et al., 2005) highlighted a significant enrichment of senescence-specific p53 (p53 enrichment score [ES] = 0.11, $p = 0.009$), STAT3 (ES = 0.137, $p < 0.001$), and STAT5 (ES = 0.084, $p = 0.039$) targets in those marker genes (Figure 2C). No enrichment of apoptosis (no values returned; data not shown) or checkpoint-related p53 targets ($p = 0.44$; Figure S2A) were detected. GSEA found no enrichment of p53 and STAT3/5 targets comparing pooled young and old HSCs ($p = 0.422$ for p53 apoptosis, $p = 0.65$ for p53 RIS, $p = 0.44$ for Stat5, and $p = 0.357$ for Stat3; Figure S2B; data not shown).

Third, we generated a JAK/STAT signature mimicking HSC behavior by stimulating a stem-cell-like cell line (HPC7) with thrombopoietin (TPO) for microarray analysis (Figure 2D) (Park et al., 2016). Binding of TPO to its receptor activates JAK2 and its downstream targets STAT1, -3, and -5 (Rawlings et al., 2004). GSEA highlighted a strong enrichment of TPO-regulated genes (top 100 genes, $p < 9.81e{-}10$) in the old-specific cluster (Figure 2D).

Lastly, we confirmed the old-specific cluster by indirect immunofluorescence (IF) in HSCs from young and old mice (n = 3) for p53 phosphorylated at serine 15 (pp53Ser15) and FosB phosphorylated at serine 27 (pFosBSer27). Only old HSCs showed high levels of pp53Ser15 and pFosBSer27 (Figure S2C). p53 response and senescence are associated with persistent levels of DNA damage. We stained old HSCs for p53 for gH2AX by IF and blind scored cells with more than five gH2AX foci. We found an enrichment for gH2AX over p53-positive cells (Figure 2E; Figure S2D), providing a mechanism for p53 upregulation. We tested the gH2AX-positive old HSCs for changes to proliferation by EdU injection into mice and co-staining for gH2AXpSer139 protein using IF. EdU incorporation

OPEN ACCESS
CellPress

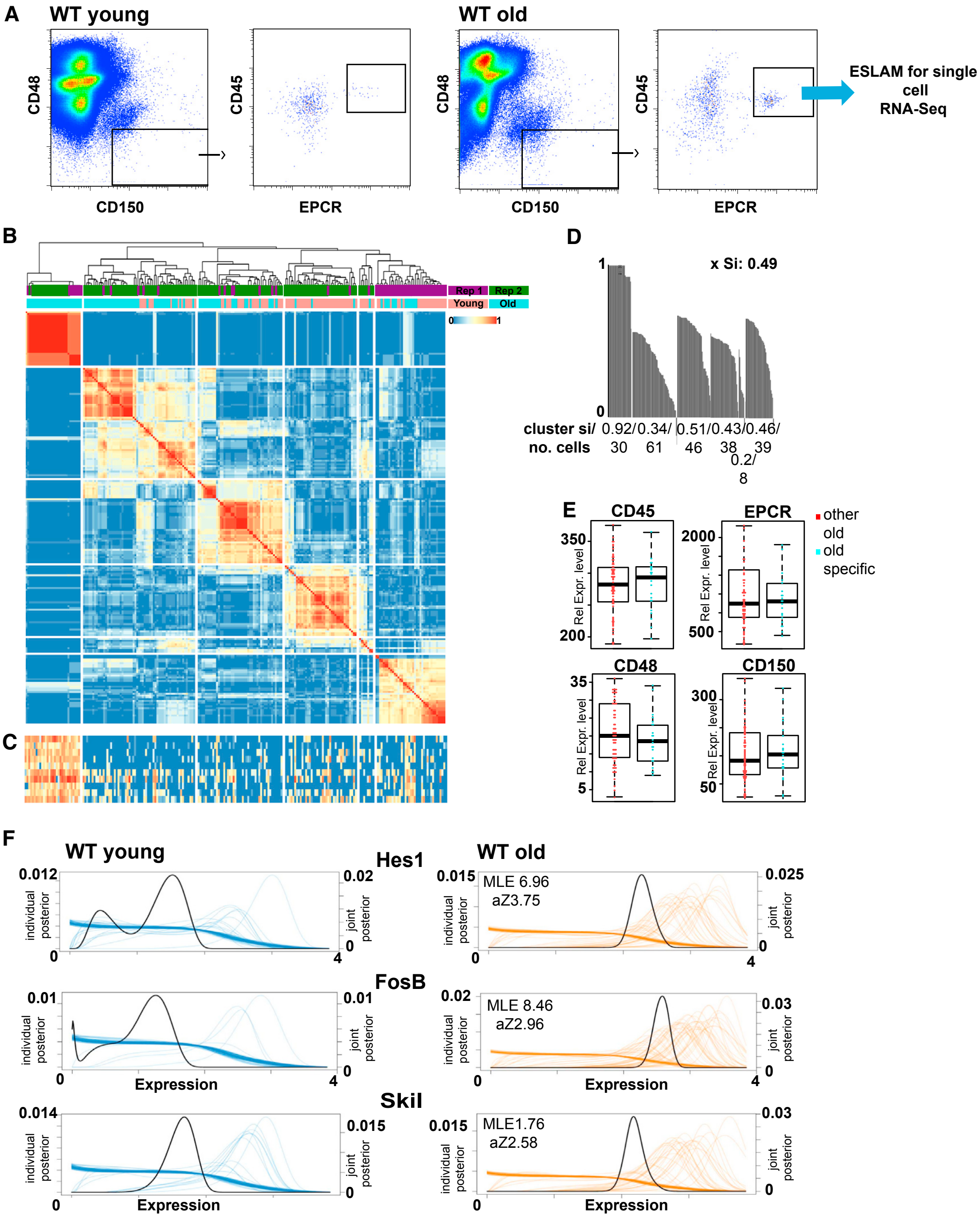

Figure 1. LT-HSCs Display a Distinct Subpopulation with Age

(A) Sorting strategy for HSCs.

(B) SC3 clustering of young and old HSC transcriptomes. Replicates: purple and green bars. Age: orange (young) and turquoise (old) bars. Similarity between cells is indicated from blue to red (identical). Rep, replicate.

(C) Heatmap of top ten marker genes of cluster 1. Expression is shown from blue (low) to red (high).

(D) Silhouette plots for all clusters. The silhouette index (si) and the number of cells per cluster are given. xSi, average silhouette index.

(E) Boxplots for ESLAM marker intensity for old-specific (blue) and other old (red) HSCs. Rel. Expr., relative expression.

(F) SCDE plots for marker genes of cluster 1 in young (blue) and old (orange) HSCs. Expression levels of individual cells are indicated by individual lines.

See also Supplemental Experimental Procedures.

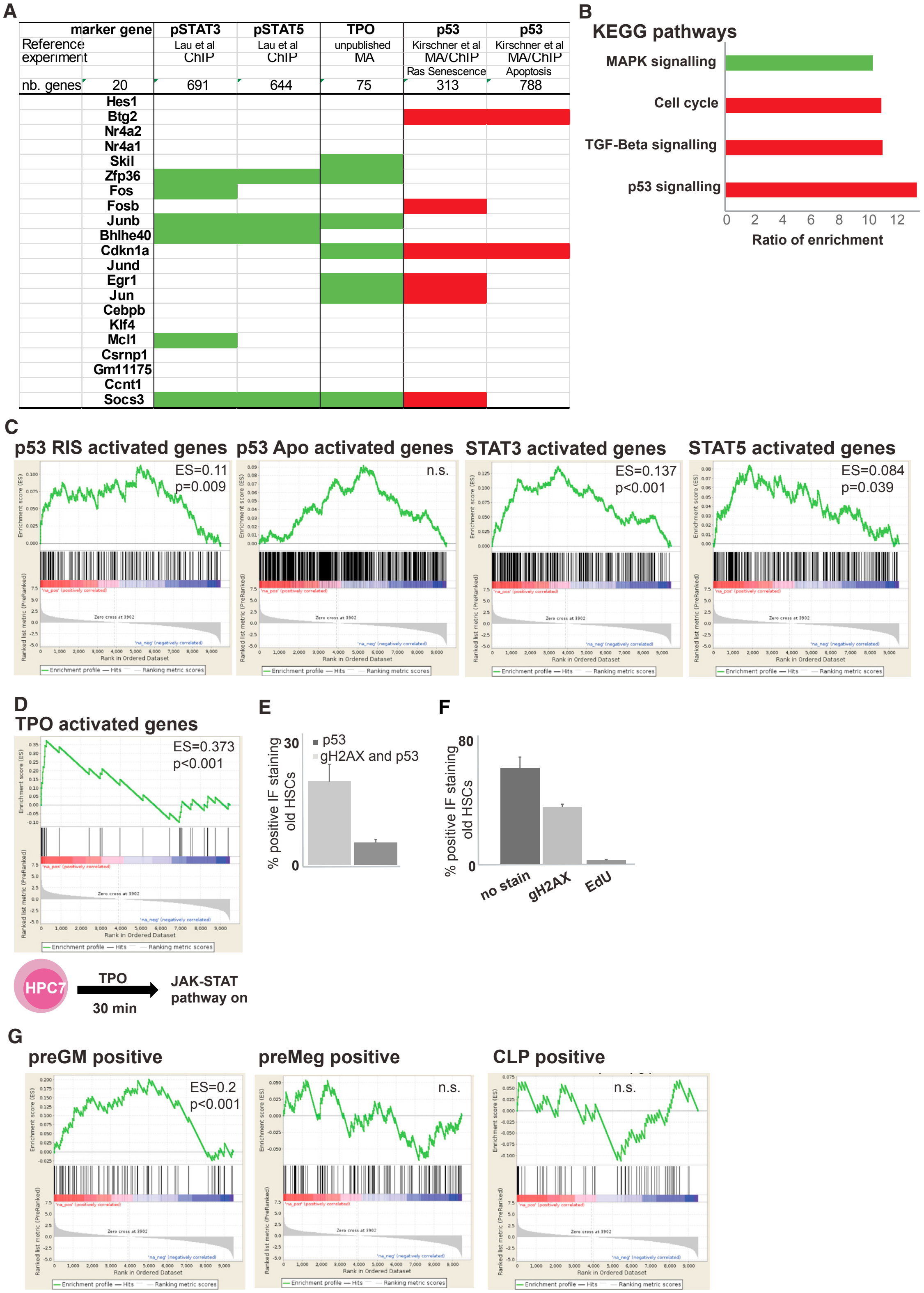

Figure 2. Age-Specific Cluster Carries Signature of Pro-proliferative and Anti-proliferative Stimuli

(A) Manual annotation of top 20 marker genes with Ras senescence (RIS), apoptosis (Kirschner et al., 2015), and pStat3 and pStat5 datasets (Lau et al., 2015). Red indicates p53, and green indicates pStat targets.

(legend continued on next page)

was absent from gH2AX-positive cells, suggesting reduced proliferation (Figure 2F).

The Old-Specific Cluster Is Enriched for Myeloid-Biased HSCs and Regulated by Transcription Factors Controlling Quiescence and Proliferation

One feature of the aging HSC compartment, the myeloid basis, has been described on a single-cell basis (Grover et al., 2016). To identify lineage biases in our old-specific cluster, we overlaid our marker gene list with gene sets for bi-potent granulocyte/macrophage progenitors (preGM), megakaryocyte/erythroid progenitors (preMeg), and common lymphoid progenitors (CLPs) using GSEA (Pronk et al., 2007). We identified an enrichment of preGM genes (ES = 0.2, $p < 0.01$), with no enrichment in preMeg or CLP genes (Figure 2G), suggesting a myeloid bias in our old-specific HSC cluster. These results could explain the myeloid bias emerging with age where a subset of myeloid-primed HSCs reconstitute the blood system more frequently over time, followed by exhaust, leaving a large pool of myeloid-primed progenitors in the system.

We interrogated the marker genes specific to our old-specific HSCs with respect to regulation of HSC function and found 8/12 transcription factors regulating quiescence and proliferation (Table S2, p = 6.779747e−24). Nuclear receptor subfamily 4 group A member 1 (Nr4a1) has been reported to be upregulated on myeloid-biased HSCs (Land et al., 2015), further supporting the myeloid bias. Perturbations of some marker genes have been reported and suggest an enrichment for quiescence regulators in the old-specific subcluster (Table S3).

Constitutive Jak2 Activation Increases Contribution to the Age-Specific Subpopulation of LT-HSCs

The upregulation of the p53 pathway in Jak2 context has previously been reported in erythroblasts from patients with myeloproliferative disease (MPN) (Chen et al., 2014). In the majority of MPNs, Jak2 is constitutively active through a mutation leading to JAK2V617F (Baxter et al., 2005), where p53 is thought to maintain genome stability in the chronic phase of disease (Chen et al., 2014). In this study, we did not observe activation of p53 in proliferating young HSCs (Figures 1C and S1C). One explanation for the lack of p53 activation in young HSCs is that p53 upregulation correlates with number of replications, similar to the role of p53 in replicative senescence (Beauséjour et al., 2003). To test the association between JAK/STAT-driven proliferation and p53 activation, we used single-cell approaches in homozygous JAK2V617F mice, where JAK2V617F provides a constant proliferative stimulus (Li et al., 2014). We first characterized proliferation kinetics of single young and old wild-type (WT) and JAK2V617F HSCs in vitro and found a significant increase in proliferation in young JAK2V617F HSCs after 72 hr in culture (p = 0.002, first division; p = 0.0001, second division; p = 0.0001, third division) (Figures 3A and 3B). Consistent with our transcriptomic data (Figure 3C), we failed to detect p53 activation in young JAK2V617F HSCs by IF for pp53Ser15 (Figure 3D), concluding that JAK2V617F increases proliferation without evoking a p53 response in young LT-HSCs. We tested whether JAK2V617F exerts an effect with age through a prolonged, lifelong increase in proliferation. We found two lines of evidence agreeing with prolonged, and not acutely enforced, proliferation evoking the old-associated p53 signature. First, we characterized the proliferation kinetics of single old JAK2V617F HSCs in vitro where old JAK2V617F HSCs show a loss of JAK2V617F-driven increased proliferation as measured by numbers of division at 72 hr in culture (p = 0.2117, first division; p = 0.0046, second division; p = 0.0039, third division). In agreement with published data in heterozygous JAK2V617F context (Kent et al., 2013), old homozygous JAK2V617F HSCs show a significant decrease in average division when compared to old WT HSCs at 48 and 72 hr in culture (48 hr: p = 0.007, first division; p = 0.0001, second division; p = 0.106, third division; 72 hr: p = 0.0338, first division; p = 0.0004, second division; p = 0.2142, third division) (Figures 3A and 3B). In summary, the JAK2V617F-enhanced proliferative effect seen in young HSCs is lost in old HSCs.

Second, to establish causality for Jak2 driving the old-specific cluster formation, we tested the effect of the long-term increase in proliferation on individual cells in the aging HSC compartment using single-cell transcriptomics. We generated single-cell transcriptional profiles of young (n = 70) and old (n = 139) LT-HSCs homozygous for JAK2V617F (Li et al., 2014). Following clustering by SC3 (Kiselev et al., 2017), we identified the same old-specific cluster as in the WT cells, evidenced by an identical set of marker genes (Figure 3E). A careful comparison of the number of cells in the old-specific cluster showed that it contained 26% and 33% of WT cells in replicates 1 and 2 respectively (Figure 3E; data not shown). In the JAK2V617V homozygous context, this contribution increased to 32% and 47% for replicates 1 and 2, respectively (Figure 3E; data not shown), a statistically non-significant increase (p = 0.146). To optimize the number of cells included in our analysis, we scored 1,463 cells from 3 old and 3 young WT and JAK2V617F mice (Figure 3D), by positive pp53Ser15 and pFosB IF. In old WT LT-HSCs, we found an increase of 36% in double-positive HSCs when compared to young WT HSCs, an increase consistent with our scRNA-seq data. The old-specific cluster expanded to 64% in JAK2V617F HSCs, consisting of significant increase compared to WT (p = 0.0001). To pinpoint the onset of the p53 signature in LT-HSCs, we generated single-cell transcriptional profiles of 12-month-old

(B) KEGG pathway analysis of marker genes for old-specific cluster. Selected pathways are shown as ratio of enrichment (green indicates pro-proliferative, and red indicates anti-proliferative).
(C) GSEA for p53 RIS, apoptosis (Apo), and STAT3 and STAT5 targets. p values and enrichment scores are shown.
(D) Schematic of TPO-regulated microarray experiment in HPC7. GSEA of TPO-specific targets and marker gene list.
(E) IF quantification of old HSCs for p53Ser15 and/or gH2AX (n = 3; error bars indicate SEM).
(F) Quantification of EdU (5-ethynyl-2′-deoxyuridine) incorporation, gH2AX, and p53 in old HSCs (n = 3; error bars indicates SEM).
(G) GSEA for lineage markers.
ChIP, chromatin immunoprecipitation, MA, microarray; TGF, transforming growth factor; n.s., not significant; nb, number of genes.

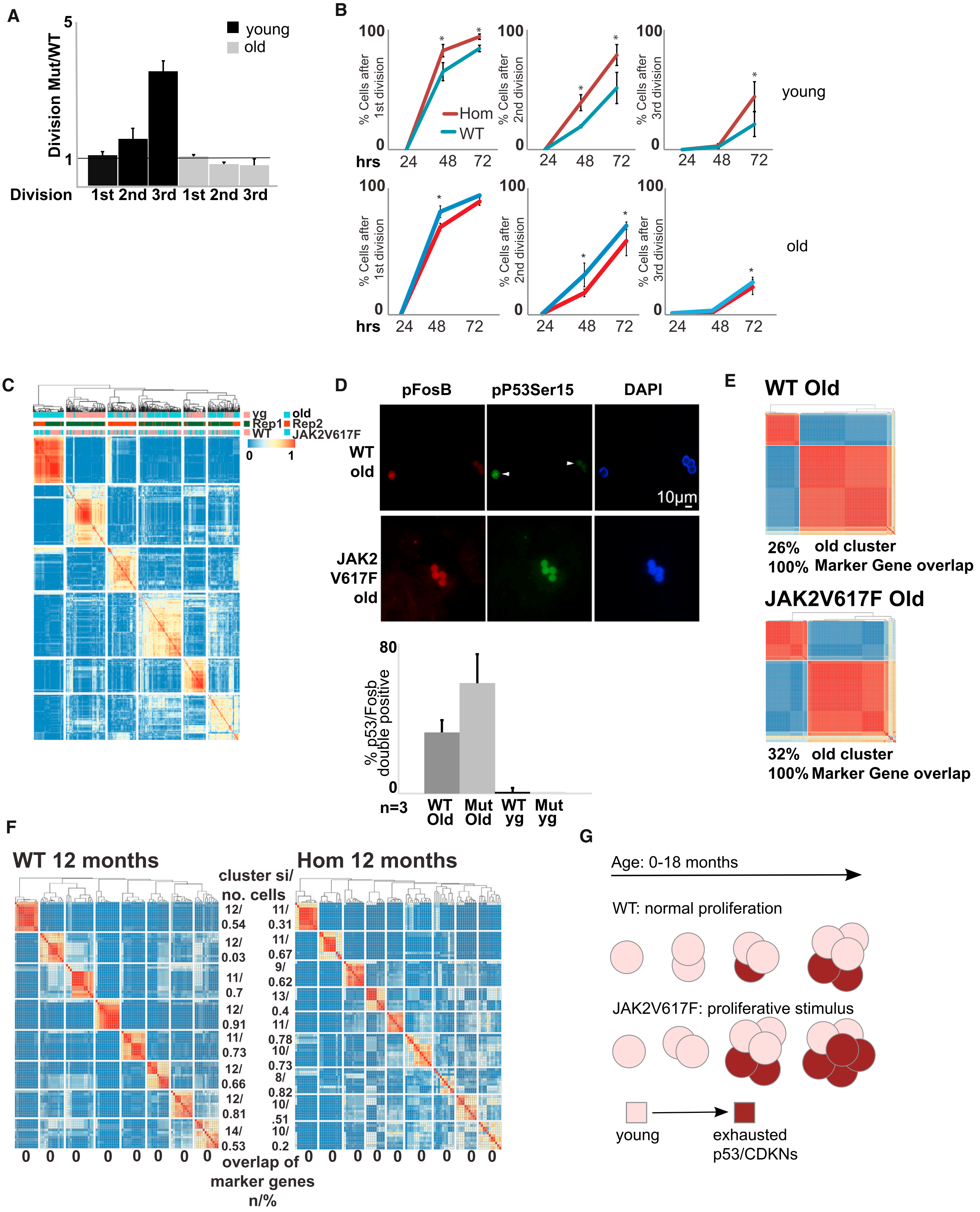

(legend on next page)

JAK2V617F mice (n = 2; 130 cells) and WT mice (n = 2; 144 cells) (Figure 3F). No overlap with the marker genes for the p53 cluster was found, suggesting that the onset of the p53 signature within LT-HSCs might be late in the mouse lifespan and a state switch-like, stochastic event. Taken together, our data suggest that pro-proliferating constitutive Jak2 activation expands the old-specific cluster, evoking a p53 response (Figure 3G). We hypothesize that enforced proliferation in young HSCs leads to stem cell exhaust and p53 activation with age.

DISCUSSION

The regenerative capacity of a tissue declines with age. Whether this reduction of stemness is equally distributed between stem cells or affects only a subset of cells is not well understood. In this study, only a distinct subpopulation of HSCs carried a signature indicative of functional decline as characterized by p53 signaling, pointing toward functionally heterogeneous stem cell aging. Whether this heterogeneous decline in function serves as a strategy to ensure stem cell function over an entire lifespan is unclear.

A role for p53 in hematopoietic aging has been established. In HSCs, increased p53 activity leads to reduced functionality and proliferation with age, whereas decreased p53 levels increase HSC proliferative capacity with age (Dumble et al., 2007), suggesting a direct link between p53 dosage and regenerative capacity in HSCs. Despite the wealth of phenotypical characterization of p53 dosage on the HSC compartment as a whole, our single-cell approach reveals how p53 activation is distributed within the HSC compartment and provides the missing deconvoluted transcriptional signatures of p53-activated and unactivated aged HSCs. Observations from studies in progeroid and hyperactivated p53 mouse models suggest a more generalized role for p53, possibly extending the relevance of our findings to non-hematopoietic tissues (Varela et al., 2005; Tyner et al., 2002). We found that p53-activated HSCs co-expressed cell-cycle inhibitory and proliferative transcripts from MAPK and JAK/STAT signaling. This co-activation of pro- and anti-proliferative pathways has been described in senescent cells induced by hyperactivation of MAPK signaling (Serrano et al., 1997). A role for JAK2 signaling has previously been implicated in the senescence induction of hematopoietic-lineage-negative $Sca^{+}c\text{-}Kit^{+}$ cells (Li et al., 2010) with accumulation of γH2Ax foci, reduced rates of proliferation, and inhibition of apoptosis. These phenomena were only observed 26 weeks after JAK2V617F induction, suggesting a cumulative rather than an immediate effect of JAK2V617F on proliferation (Li et al., 2010). A link between enforced proliferation and stem cell exhaustion has been shown where loss of CDKN1A drives HSCs into hyperproliferation and subsequent exhaust (Cheng et al., 2000). A recent study showed number of cell divisions as a limiting factor in HSC regenerative capacity (Bernitz et al., 2016). Based on the co-activation of anti-proliferative pathways of p53 in individual HSCs, the opposing effects of JAK2V617F on HSC proliferation in young and old mice and the increase of p53-activated cells under prolonged, but not acute, JAK2V617F activation, we provide support for a link between cumulative proliferation and stem cell exhaustion with age.

We identified a myeloid bias in the old-specific HSC population that could not be detected in the other old HSCs (Figure 2F). Myeloid bias is well documented in the literature (Rossi et al., 2005), but molecular mechanisms are, thus far, elusive. We speculate that myeloid-biased HSCs proliferate more over the lifetime of a mouse, leading to HSC decline at the same time as providing a pool of myeloid-biased progenitors reconstituting the HSC compartment. This heterogeneity in HSC aging might be mediated by the bone marrow niche, where it has been shown that aged HSCs home further away from the endosteum compared to young HSCs, leading to decreased regenerative capacity (Geiger et al., 2013). However, this hypothesis awaits further investigation.

EXPERIMENTAL PROCEDURES

Mice

Mice were generated as described previously (Li et al., 2014). All mice were kept in specific pathogen-free conditions and all procedures were performed according to rules of the Animal Welfare and Ethical Review Body (AWERB) and the UK Home Office regulations.

Cell Isolation and Flow Cytometry

Single cells were obtained from bone marrow suspensions as described previously (Kent et al., 2013). ESLAM cells were isolated using CD45-FITC (clone 30-F11), EPCR-PE (clone RMEPCR1560), CD150-Pacific Blue (clone TC15), and CD48-APC (clone HM48-1).

Figure 3. Constitutively Active JAK2 Increases Cell Contribution to the Old-Specific Subpopulation

(A) Kinetics of JAK2V617F and WT HSCs. Ratios of JAK2V617F and WT for indicated divisions in young (black) and old (gray) HSCs. Black line denotes WT levels (n = 3; error bars indicate SEM). Mut, mutated.

(B) Kinetics data from young (upper row) and old (lower row) WT (blue) and homozygous (Hom) JAK2V671F (red) HSCs. Percentage of all cells upon division (n = 3; error bars indicate SEM). hrs, hours.

(C) SC3 clustering of HSC transcriptomes from young (yg; upper orange bars) and old (upper turquoise bars) WT (lower orange bars) and JAK2V617F (lower turquoise bars) mice. Replicates are indicated in green and red. Similarity between cells is indicated from blue to red (identical).

(D) SC3 clustering of old HSC transcriptomes from WT and JAK2V617F mice (replicate 1). Overlap with marker genes is given as a percentage. Cell number for old-specific HSCs is given as a percentage of all old HSCs.

(E) Immunofluorescent images of old WT and JAK2V617F HSCs stained for pSer15p53 (green), pSer27FosB (red), and DAPI (blue). Bar plots quantify the percentage of double-positive HSCs from indicated mice (n = 3; error bars indicate SEM).

(F) SC3 clustering of 1-year-old WT and JAK2V617F HSCs. Similarity between cells is indicated from blue to red (identical). Overlap with marker genes is shown in percentages.

(G) Model for relation between proliferation and functional decline of HSCs. Compared are physiological (upper row) against enforced proliferation through JAK2V617F (lower row). Young HSCs in the JAK2 condition expand faster (number of cells) but exhaust more readily upregulating p53- and cyclin-dependent kinase inhibitors (CDKNs; shades of red). Arrow cycle indicates self-renewal.

Single-Cell cDNA and Library Preparation

cDNA from single cells was obtained as described (Picelli et al., 2013). Illumina Nextera reagents were used for library construction and sequenced on the HiSeq 2500 Sequencing System (125-bp paired-end [PE] reads).

Data Analysis

Data analysis was performed using SC3 and SCDE tools (Kiselev et al., 2017; Kharchenko et al., 2014). For details, see the Supplemental Experimental Procedures.

IF

ESLAM cells were processed as described previously (Li et al., 2010), using anti-p-Serine15 p53 (clone 16G8), anti-p-Serine139 gH2AX (JBW39), and anti-p-Serine27 FosB (ab 62433) antibodies. Cells were blinded and scored on an AxioImager Z2 (Zeiss). A two-sided Fisher's exact test was used to calculate p values.

Single ESLAM Cultures

Single ESLAM cells were cultured, blinded, and counted as described previously (Kent et al., 2013). A two-sided Fisher's exact test was used to calculate the p values.

HPC7 Cell Culture and Microarray

HPC7 cells were grown as described previously (Park et al., 2016). For microarray, cells were serum starved and stimulated with TPO for 30 min. Total RNA was extracted using the RNeasy Mini Kit (QIAGEN). Cambridge Genomic Services processed all samples using Illumina WG-6 BeadArrays. For data analysis, see Supplemental Experimental Procedures.

ACCESSION NUMBERS

The accession number for the FACS data reported in this paper is FlowRepository: FR-FCM-ZY64. The accession numbers for the microarray and single-cell sequencing data reported in this paper are GEO: GSE87687 and GSE87631.

SUPPLEMENTAL INFORMATION

Supplemental Information includes Supplemental Experimental Procedures, two figures, and three tables and can be found with this article online at http://dx.doi.org/10.1016/j.celrep.2017.04.074.

AUTHOR CONTRIBUTIONS

K.K., T.C., W.R., and A.R.G. designed the study. K.K., T.C., I.C.M., H.J.P., J.L., D.G.K., and R.K. performed experiments. D.F.-S.C., V.K., M.H., and T.C. performed bioinformatics analysis. K.K. analyzed all other data. T.L.H. and D.C.P. helped with mouse experiments. K.K., T.C., W.R., and A.R.G. wrote the manuscript.

ACKNOWLEDGMENTS

We would like to thank the CIMR Flow Cytometry Core Facility, especially Dr. Chiara Cosetti and Dr. Reiner Schulte. The laboratory of Wolf Reik is supported by the BBSRC (BB/K010867/1), the Wellcome Trust (095645/Z/11/Z), EU BLUEPRINT, and EpiGeneSys. Work in the A.R.G. lab is supported by Bloodwise (grant ref. 13003), the Wellcome Trust (grant ref. 104710/Z/14/Z), the Medical Research Council, the Kay Kendall Leukaemia Fund, the Cambridge NIHR Biomedical Research Center, the Cambridge Experimental Cancer Medicine Centre, the Leukemia and Lymphoma Society of America (grant ref. 07037), and a core support grant from the Wellcome Trust and MRC to the Wellcome Trust-Medical Research Council Cambridge Stem Cell Institute. K.K. is supported by a Wellcome Trust ISSF Fellowship (grant 105641/Z/14/Z). The M.H. lab is supported by a Wellcome Trust core grant to the WTSI. T.C. is supported by a Chancellor's Fellowship held at the University of Edinburgh. W.R. is a consultant and shareholder of Cambridge Epigenetix.

Received: October 15, 2016
Revised: March 21, 2017
Accepted: April 26, 2017
Published: May 23, 2017

REFERENCES

Baxter, E.J., Scott, L.M., Campbell, P.J., East, C., Fourouclas, N., Swanton, S., Vassiliou, G.S., Bench, A.J., Boyd, E.M., Curtin, N., et al. (2005). Acquired mutation of the tyrosine kinase JAK2 in human myeloproliferative disorders. Lancet *365*, 1054–1061.

Beauséjour, C.M., Krtolica, A., Galimi, F., Narita, M., Lowe, S.W., Yaswen, P., and Campisi, J. (2003). Reversal of human cellular senescence: roles of the p53 and p16 pathways. EMBO J. *22*, 4212–4222.

Beckerman, R., and Prives, C. (2010). Transcriptional regulation by p53. Cold Spring Harb. Perspect. Biol. *2*, a000935.

Beerman, I., Maloney, W.J., Weissmann, I.L., and Rossi, D.J. (2010). Stem cells and the aging hematopoietic system. Curr. Opin. Immunol. *22*, 500–506.

Bernitz, J.M., Kim, H.S., MacArthur, B., Sieburg, H., and Moore, K. (2016). Hematopoietic stem cells count and remember self-renewal divisions. Cell *167*, 1296–1309.e10.

Chen, E., Ahn, J.S., Massie, C.E., Clynes, D., Godfrey, A.L., Li, J., Park, H.J., Nangalia, J., Silber, Y., Mullally, A., et al. (2014). JAK2V617F promotes replication fork stalling with disease-restricted impairment of the intra-S checkpoint response. Proc. Natl. Acad. Sci. USA *111*, 15190–15195.

Cheng, T., Rodrigues, N., Shen, H., Yang, Y., Dombkowski, D., Sykes, M., and Scadden, D.T. (2000). Hematopoietic stem cell quiescence maintained by p21cip1/waf1. Science *287*, 1804–1808.

Dumble, M., Moore, L., Chambers, S.M., Geiger, H., Van Zant, G., Goodell, M.A., and Donehower, L.A. (2007). The impact of altered p53 dosage on hematopoietic stem cell dynamics during aging. Blood *109*, 1736–1742.

Dykstra, B., Olthof, S., Schreuder, J., Ritsema, M., and de Haan, G. (2011). Clonal analysis reveals multiple functional defects of aged murine hematopoietic stem cells. J. Exp. Med. *208*, 2691–2703.

Geiger, H., de Haan, G., and Florian, M.C. (2013). The ageing haematopoietic stem cell compartment. Nat. Rev. Immunol. *13*, 376–389.

Grover, A., Sanjuan-Pla, A., Thongjuea, S., Carrelha, J., Giustacchini, A., Gambardella, A., Macaulay, I., Mancini, E., Luis, T.C., Mead, A., et al. (2016). Single-cell RNA sequencing reveals molecular and functional platelet bias of aged haematopoietic stem cells. Nat. Commun. *7*, 11075.

Kanehisa, M., Goto, S., Sato, Y., Furumichi, M., and Tanabe, M. (2012). KEGG for integration and interpretation of large-scale molecular data sets. Nucleic Acids Res. *40*, D109–D114.

Kent, D.G., Li, J., Tanna, H., Fink, J., Kirschner, K., Pask, D.C., Silber, Y., Hamilton, T.L., Sneade, R., Simons, B.D., and Green, A.R. (2013). Self-renewal of single mouse hematopoietic stem cells is reduced by JAK2V617F without compromising progenitor cell expansion. PLoS Biol. *11*, e1001576.

Kenzelmann Broz, D., Spano Mello, S., Bieging, K.T., Jiang, D., Dusek, R.L., Brady, C.A., Sidow, A., and Attardi, L.D. (2013). Global genomic profiling reveals an extensive p53-regulated autophagy program contributing to key p53 responses. Genes Dev. *27*, 1016–1031.

Kharchenko, P.V., Silberstein, L., and Scadden, D.T. (2014). Bayesian approach to single-cell differential expression analysis. Nat. Methods *11*, 740–742.

Kirschner, K., Samarajiwa, S.A., Cairns, J.M., Menon, S., Pérez-Mancera, P.A., Tomimatsu, K., Bermejo-Rodriguez, C., Ito, Y., Chandra, T., Narita, M., et al. (2015). Phenotype specific analyses reveal distinct regulatory mechanism for chronically activated p53. PLoS Genet. *11*, e1005053.

Kiselev, V.Y., Kirschner, K., Schaub, M.T., Andrews, T., Yiu, A., Chandra, T., Natarajan, K.N., Reik, W., Barahona, M., Green, A.R., and Hemberg, M. (2017). SC3: consensus clustering of single-cell RNA-seq data. Nat. Methods *14*, 483–486.

Kowalczyk, M.S., Tirosh, I., Heckl, D., Rao, T.N., Dixit, A., Haas, B.J., Schneider, R.K., Wagers, A.J., Ebert, B.L., and Regev, A. (2015). Single-cell RNA-seq reveals changes in cell cycle and differentiation programs upon aging of hematopoietic stem cells. Genome Res. *25*, 1860–1872.

Land, R.H., Rayne, A.K., Vanderbeck, A.N., Barlowe, T.S., Manjunath, S., Gross, M., Eiger, S., Klein, P.S., Cunningham, N.R., Huang, J., et al. (2015). The orphan nuclear receptor NR4A1 specifies a distinct subpopulation of quiescent myeloid-biased long-term HSCs. Stem Cells *33*, 278–288.

Lau, W.W.Y., Hannah, R., Green, A.R., and Göttgens, B. (2015). The JAK-STAT signaling pathway is differentially activated in CALR-positive compared with JAK2V617F-positive ET patients. Blood *125*, 1679–1681.

Li, J., Spensberger, D., Ahn, J.S., Anand, S., Beer, P.A., Ghevaert, C., Chen, E., Forrai, A., Scott, L.M., Ferreira, R., et al. (2010). JAK2 V617F impairs hematopoietic stem cell function in a conditional knock-in mouse model of JAK2 V617F-positive essential thrombocythemia. Blood *116*, 1528–1538.

Li, J., Kent, D.G., Godfrey, A.L., Manning, H., Nangalia, J., Aziz, A., Chen, E., Saeb-Parsy, K., Fink, J., Sneade, R., et al. (2014). JAK2V617F homozygosity drives a phenotypic switch in myeloproliferative neoplasms, but is insufficient to sustain disease. Blood *123*, 3139–3151.

Nestorowa, S., Hamey, F.K., Pijuan Sala, B., Diamanti, E., Shepherd, M., Laurenti, E., Wilson, N.K., Kent, D.G., and Göttgens, B. (2016). A single-cell resolution map of mouse hematopoietic stem and progenitor cell differentiation. Blood *128*, e20–e31.

Park, H.J., Li, J., Hannah, R., Biddie, S., Leal-Cervantes, A.I., Kirschner, K., Flores Santa Cruz, D., Sexl, V., Göttgens, B., and Green, A.R. (2016). Cytokine-induced megakaryocytic differentiation is regulated by genome-wide loss of a uSTAT transcriptional program. EMBO J. *35*, 580–594.

Picelli, S., Björklund, Å.K., Faridani, O.R., Sagasser, S., Winberg, G., and Sandberg, R. (2013). Smart-seq2 for sensitive full-length transcriptome profiling in single cells. Nat Methods. *10*, 1096–1098.

Pronk, C.J.H., Rossi, D.J., Månsson, R., Attema, J.L., Norddahl, G.L., Chan, C.K., Sigvardsson, M., Weissman, I.L., and Bryder, D. (2007). Elucidation of the phenotypic, functional, and molecular topography of a myeloerythroid progenitor cell hierarchy. Cell Stem Cell *1*, 428–442.

Rawlings, J.S., Rosler, K.M., and Harrison, D.A. (2004). The JAK/STAT signaling pathway. J. Cell Sci. *117*, 1281–1283.

Rossi, D.J., Bryder, D., Zahn, J.M., Ahlenius, H., Sonu, R., Wagers, A.J., and Weissman, I.L. (2005). Cell intrinsic alterations underlie hematopoietic stem cell aging. Proc. Natl. Acad. Sci. USA *102*, 9194–9199.

Rossi, D.J., Bryder, D., Seita, J., Nussenzweig, A., Hoeijmakers, J., and Weissman, I.L. (2007). Deficiencies in DNA damage repair limit the function of haematopoietic stem cells with age. Nature *447*, 725–729.

Serrano, M., Lin, A.W., McCurrach, M.E., Beach, D., and Lowe, S.W. (1997). Oncogenic ras provokes premature cell senescence associated with accumulation of p53 and p16INK4a. Cell *88*, 593–602.

Subramanian, A., Tamayo, P., Mootha, V.K., Mukherjee, S., Ebert, B.L., Gillette, M.A., Paulovich, A., Pomeroy, S.L., Golub, T.R., Lander, E.S., and Mesirov, J.P. (2005). Gene set enrichment analysis: a knowledge-based approach for interpreting genome-wide expression profiles. Proc. Natl. Acad. Sci. USA *102*, 15545–15550.

Tyner, S.D., Venkatachalam, S., Choi, J., Jones, S., Ghebranious, N., Igelmann, H., Lu, X., Soron, G., Cooper, B., Brayton, C., et al. (2002). p53 mutant mice that display early ageing-associated phenotypes. Nature *415*, 45–53.

Vainchenker, W., and Constantinescu, S.N. (2013). JAK/STAT signaling in hematological malignancies. Oncogene *32*, 2601–2613.

Varela, I., Cadiñanos, J., Pendás, A.M., Gutiérrez-Fernández, A., Folgueras, A.R., Sánchez, L.M., Zhou, Z., Rodríguez, F.J., Stewart, C.L., Vega, J.A., et al. (2005). Accelerated ageing in mice deficient in Zmpste24 protease is linked to p53 signalling activation. Nature *437*, 564–568.

Cell Reports, Volume *19*

Supplemental Information

Proliferation Drives Aging-Related Functional Decline in a Subpopulation of the Hematopoietic Stem Cell Compartment

Kristina Kirschner, Tamir Chandra, Vladimir Kiselev, David Flores-Santa Cruz, Iain C. Macaulay, Hyun Jun Park, Juan Li, David G. Kent, Rupa Kumar, Dean C. Pask, Tina L. Hamilton, Martin Hemberg, Wolf Reik, and Anthony R. Green

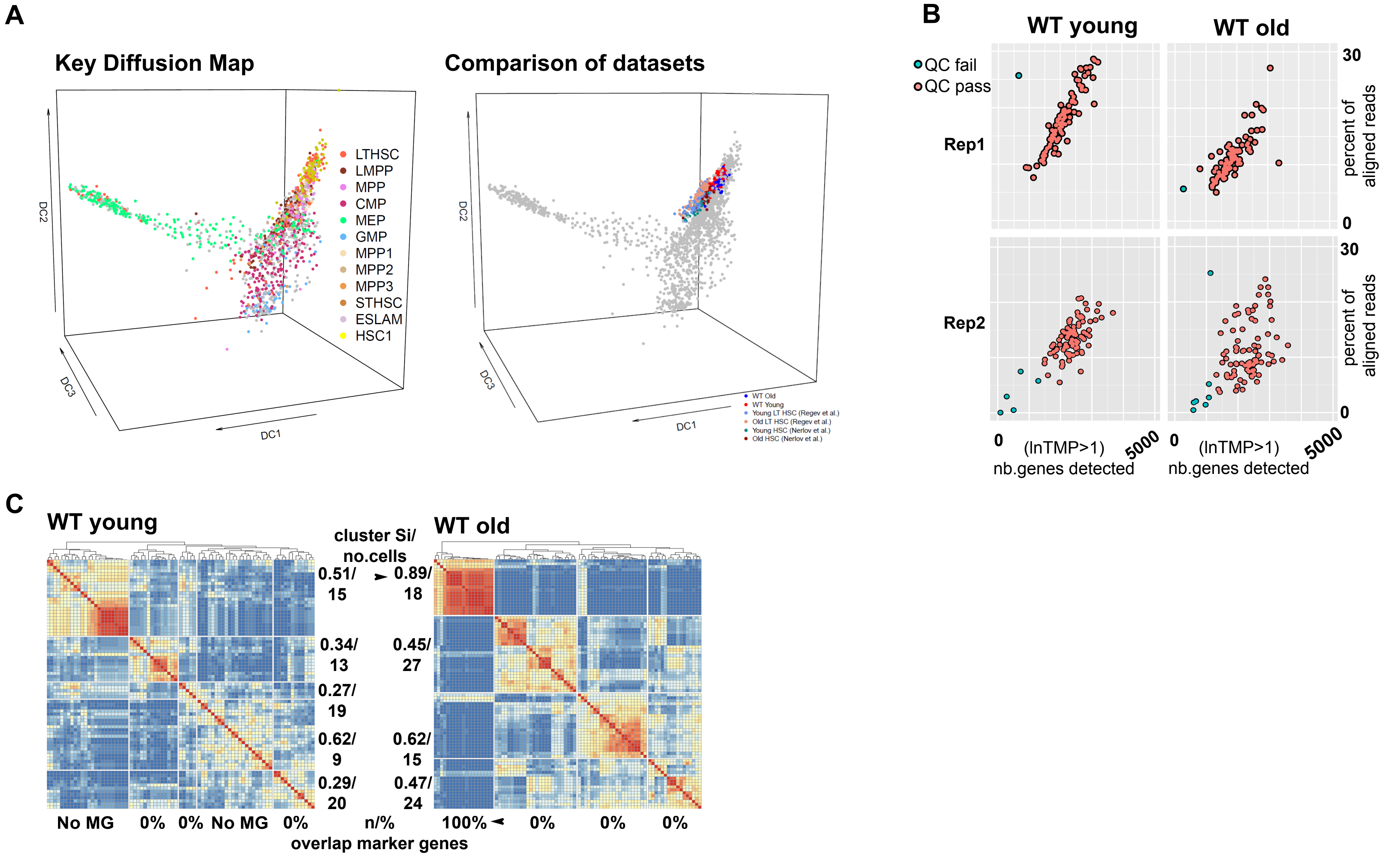

Supplemental Figure 1: Additional quality control and analysis of single cell RNA-Seq data, Related to Figure 1:
A) Diffusion map defining the single cell transcriptome of several murine haematopoietic cell types as indicated on left hand side (Nestorowa et al., 2016). Our HSC data alongside the HSC data from Grover and colleagues (Grover et al., 2016) and Regev group (Kowalczyk et al., 2015) were then superimposed onto the diffusion map (grey cells, right hand side). Colours indicate the cell types on left hand side as specified in the legend and data sets on right hand side. B) Quality control plots showing individual cells failing (green) and passing (red) the test. The percentage of aligned reads over the number of genes detected is shown with a cut off of 1300 genes. TMP transcript per million. C) Heat maps of all young or all old HSCs using SC3 unsupervised clustering tool. The intensity of the cluster is indicated from 0 (blue, not upregulated) to 1 (red, upregulated). The silhouette index (si) is given for each cluster. The overlap with marker genes identified in Fig.1c is given as percentage of all cells analysed for each age group. No MG denotes a lack of marker genes for a given cluster.

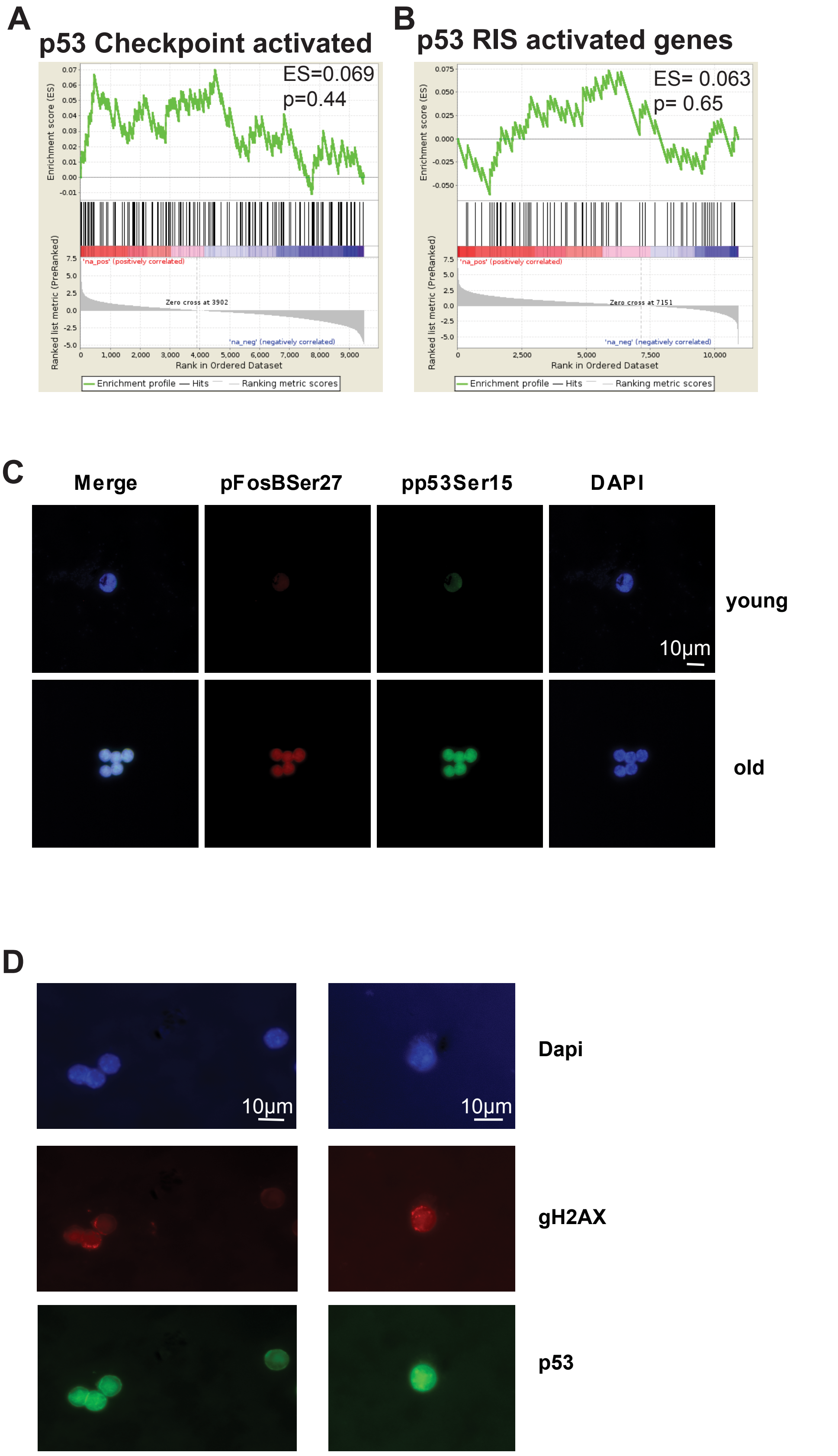

Supplemental Figure 2: Additional data supporting p53 upregulation in old specific cluster only, Related to Figure 2:
A) Gene Set enrichment plot (Subramanian et al., 2005) comparing p53 checkpoint related target genes with differentially expressed genes between the old specific cluster and all other old HSCs. Adjusted p-value and enrichment score are indicated. B) Gene Set enrichment plot (Subramanian et al., 2005) comparing p53 RIS target genes with differentially expressed genes between pooled young and old WT HSCs. Adjusted p-value and enrichment score are indicated. C) Immunofluorescence images of young and old HSCs stained for p53 phosphorylated at Serine 15 (green) and gH2AX phosphorylated at Serine 139 (red). DAPI stained nuclei are blue. Representative images are shown. D) Immunofluorescence images of old HSCs stained for p53 phosphorylated at Serine 15 (green) and FosB phosphorylated at Serine 27 (red). DAPI stained nuclei are blue. Representative images are shown.

Supplemental methods

Flow cytometry

All samples were index sorted on a BD Influx with filters and five lasers. Single ESLAM cells were either sorted into round bottom 96 well plates preloaded with serum-free medium for kinetics experiments or into 96 well PCR plates containing a mild lysis buffer with ribonuclease inhibitor as described for single cell RNA-Sequencing (Picelli et al., 2013). For Immunofluorescence experiments, ESLAM cells were sorted into tubes and mounted onto coated Cytoslides (Thermo Scientific).

Data analysis

96 single cells samples were sequenced, returning a variable number of reads (μ = 2, 017, 758 σ = 1, 886, 443). RNA-Seq by Expectation Maximisation) (RSEM) (Li and Dewey, 2011) which implements an Expectation Maximisation algorithm that allows computing the maximum likelihood of the estimate by combining information from both unique and multi mapped reads was used to estimate number of transcripts and transcripts per million (TPM) per gene. Parallelization of the alignment was accomplished with GNU Parallel ("GNU Parallel: The Command-Line Power Tool | USENIX," n.d.). The reads were mapped against the Ensembl mouse reference genome version GRC3877 (Kersey et al., 2016) with the inclusion of the reference for the spike in controls from the ERCC consortium (Baker et al., 2005) using Tophat (Trapnell et al., 2009). TPM estimates were used for quality control of the cells similarly to Kumar and colleagues (Kumar et al., 2014). Cells with less than 2500 detected transcripts were removed from posterior analysis. The SCDE R package from Kharchenko Lab (Kharchenko et al., 2014) provides a method for the analysis of differential expression in single cells. Estimate counts per gene previously calculated with RSEM (Li and Dewey, 2011) were used to compute the differential expression, genes with insufficient number of reads or not seen in enough cells were removed. Individual posterior shows the likelihood of differential expression for each cell. In Figure 1F) SCDE plots for marker genes of cluster 1 in young (blue) and old (orange) HSCs are shown. Expression levels of individual cells are given by individual lines. Individual posterior shows the likelihood of differential expression for each cell. Joint posterior depicts the likelihood of differential expression of a group of cells within the same condition. The black line indicates the estimated joint posterior distribution for the overall level for each cell type. MLE denotes the likelihood of differential expression between two conditions and adjusted Z-scores are given for indicated genes comparing two conditions.

RNA-Seq data were clustered and marker genes obtained by using SC3 (Kiselev et al., 2016). KEGG pathway analysis (Kanehisa et al., 2012) was performed using Webgestalt (Wang et al., 2013). Entrez Genes were annotated and pathway analysis conducted using hypergeometric test with multiple test adjustment (BH) and significance level of Top10. A minimum of 2 genes per category needed to be present. Enrichment analysis was performed using Gene Set Enrichment Analysis (GSEA) (Subramanian et al., 2005). The z-score of the expression difference provided by the SCDE package (Kharchenko et al., 2014) between old specific and all other old WT HSCs (p53, Stat3, Stat5, TPO and Lineage markers, see Figure 2C and 2G) or between young WT and Jak2V617F HSCs was used as a rank list of genes to compare against a defined set of genes as a background (p53, Stat3, Stat5, Sup. Fig. 2B). The significance of the overlap between marker genes and p53 targets or Stat3 and Stat5 was calculated using the hypergeometric test. The p-value was calculated assuming a total of m white balls and 20000-m black balls where a total of 20 balls were drawn. Here, m is either 313 or 768 and the number of white balls obtained was either 6 or 2.

For microarray analysis, raw probe-set intensities were processed with the R package lumi (Du et al., 2008). The lumiR function allows the processing of the data with the package, while mapping the Illumina identifiers to Mus musculus RefSeq IDs and Entrez_gene_ID through the lumi MouseID Mapping package. The raw data was normalised with the lumiExpresso function (Du et al., 2008). The differentially expressed genes were identified using the limma package (Smyth, 2004).

Supplemental References

Alder, J.K., Georgantas, R.W., Hildreth, R.L., Civin, C.I., 2006. 348. Kruppel-Like Factor 4 Regulates Proliferation of Human and Mouse Hematopoietic Stem-Progenitor Cells, but Is Not Essential for Mouse Hematopoietic Repopulation. Mol. Ther. 13, S132–S133. doi:10.1016/j.ymthe.2006.08.406

Berger, I., Shaul, Y., 1998. c-Fos antagonizes the junD gene positive autoregulatory loop; a novel c-Fos role in promoter switching. Gene 211, 375–382. doi:10.1016/S0378-1119(98)00120-6

Kowalczyk, M.S., Tirosh, I., Heckl, D., Rao, T.N., Dixit, A., Haas, B.J., Schneider, R.K., Wagers, A.J., Ebert, B.L., Regev, A., 2015. Single-cell RNA-seq reveals changes in cell cycle and differentiation programs upon aging of hematopoietic stem cells. Genome Res. . doi:10.1101/gr.192237.115

Liebermann, D.A., Gregory, B., Hoffman, B., 1998. International journal of oncology., International Journal of Oncology. University of Crete, Faculty of Medicine, Laboratory of Clinical Virology.

Mayani, H., 2016. The regulation of hematopoietic stem cell populations. F1000Research 5, F1000 Faculty Rev-1524. doi:10.12688/f1000research.8532.1

Okada, S., Fukuda, T., Inada, K., Tokuhisa, T., 1999. Prolonged Expression of c-fos Suppresses Cell Cycle Entry of Dormant Hematopoietic Stem Cells. Blood 93, 816 LP-825.

Santaguida, M., Schepers, K., King, B., Sabnis, A.J., Forsberg, E.C., Attema, J.L., Braun, B.S., Passegué, E., Costa, D.B., Wagner, K., al., et, 2009. JunB protects against myeloid malignancies by limiting hematopoietic stem cell proliferation and differentiation without affecting self-renewal. Cancer Cell 15, 341–52. doi:10.1016/j.ccr.2009.02.016

Sirin, O., Lukov, G., Mao, R., Conneely, O., Goodell, M.A., 2010. The Orphan Nuclear Receptor Nurr1 Restricts the Proliferation of Hematopoietic Stem Cells. Nat. Cell Biol. 12, 1213–1219. doi:10.1038/ncb2125

Vanegas, N.-D.P., Vernot, J.-P., 2017. Loss of quiescence and self-renewal capacity of hematopoietic stem cell in an in vitro leukemic niche. Exp. Hematol. Oncol. 6, 2. doi:10.1186/s40164-016-0062-1

Yu, X., Alder, J.K., Chun, J.H., Friedman, A.D., Heimfeld, S., Cheng, L., Civin, C.I., 2006. HES1 Inhibits Cycling of Hematopoietic Progenitor Cells via DNA Binding. Stem Cells 24, 876–888. doi:10.1634/stemcells.2005-0598
